# Therapeutical and Pharmaceutical Notes

**Published:** 1899-11

**Authors:** W. J. Martin

**Affiliations:** Kankakee, Ill.


### THERAPEUTICAL AND PHARMACEUTICAL NOTES.

UNDER THE DIRECTION OF W. J. MARTIN, M.D.C.,
KANKAKEE, ILL.

FORMALIN AS AN ANTISEPTIC.—The name "formalin" is given to a 40 per cent. solution of chemically pure formaldehyde in water. Formaldehyde ($CH_2O$) is a gaseous body which is prepared by subjecting methyl-alcohol to oxidation. It is readily absorbed by water; for this reason it is put on the market in the form of a saturated aqueous solution termed "formalin." Formalin mixes with water in all proportions. It is, therefore, easy to prepare any dilution that is wanted. If a 2½ per cent. solution of formalin is desired, one part of formalin is added to forty parts of water; this produces forty-one parts of a 1 per cent. formaldehyde.

After a thorough trial, extending over a period of more than one year in quite an extensive surgical practice, the author finds that as a surgical antiseptic formalin is entitled to the utmost confidence of the surgeon. The general opinion hitherto held that this drug was non-irritant and non-corrosive in its action has of late been found to be erroneous. The drug is quite irritating to abraded tissues in solutions of concentrated strength. It is also quite corrosive to surgical instruments immersed in such solutions. In very concentrated solutions the drug is also liable to become toxic by absorption. In the preparation of the field of operation, and for the immersion of instruments during an operation, we much prefer a 5 per cent. solution of carbolic acid to formalin, on account of the latter's corrosive action upon the instruments.

It is as an after-dressing for surgical and accidental wounds that formalin has given the utmost satisfaction in our practice. The strength of the solution we use for this purpose ranges from ½ to 1 per cent. In this strength the drug is non-irritating and non-corrosive in its action, and no danger of toxic absorption need be apprehended. It is best applied in the form of a fine spray with a small syringe or atomizer several times a day. In this strength formalin is the cheapest as well as the most effective antiseptic for general veterinary practice of any drug with which we are acquainted, a pound of the 40 per cent. solution of formalin costing about 50 cents.

In the treatment of lacerated and penetrating wounds, poll-evil and fistulous wither, pyæmic infection resulting from infected wounds, etc., there is probably no drug or chemical that has such lethal action upon disease-producing organisms as has formalin. In cases of protracted and difficult parturition, so often met with in country practice, in which the practitioner is not called in until the obstructing foal or calf is already in an advanced state of decomposition, in formalin the practitioner will find a powerful protecting friend in every instance. In these cases it is well to use the drug in much stronger solution than in surgical dressings. From 4 to 5 per cent. solutions can be safely used. Formalin has secured a permanent place as the chief disinfectant in my obstetric cases, and is used by me as a preventive against septic infection in preference to other antiseptics.

In stables in which horses are suffering from acute coryza or strangles, acute catarrhal laryngitis, infectious pneumonia, etc., the spraying of such stables with dilute solutions of formalin will prove highly beneficial. From some recent experiments conducted with this drug concerning its bactericidal properties it was found[1] that a solution of formalin, 1 : 10,000, checks the development of the bacilli of anthrax, cholera, typhoid, diphtheria, and staphylococcus aureus. Very dilute vapors of formalin have the same action. A 1 per cent. solution of formalin kills pure culture of pathogenic germs in an hour. This action is most marked in dilute alcoholic solution.

Where Some Drugs Come From.—Oil of cajeput of the best variety comes from Maccassar, on the Island of Clebees, about 1500 miles due south of Manillia, in the Dutch East Indies. Cape aloes and buchu leaves of the better grades come from the Cape of Good Hope, Africa. The home of the vanilla bean is in Mexico, principally in the States of Vera Cruz, Tabasco, and in the Isthmus of Tehuantepec. The native habitat of erythroxylum coca is in the mountain provinces of Peru and Bolivia, where the use of the leaves as a cardiac and nerve stimulant was well known to the ancient Peruvians under the Inca dynasties. The principal sources of sassafras, a tree indigenous to the Western Hemisphere, is from the Southern States bordering on the Atlantic Ocean. Some idea of the vast amount of sassafras root consumed in commerce may be gained from a recent statement that in one county in Virginia 80,000 pounds of sassafras wood was con-

[1] Medical News.

sumed daily in distillation, from which was obtained about fifty gallons of oil.

Adulteration of Drugs.—In a paper read before the Illinois Pharmaceutical Society, Prof. Davoll, of the Northwestern School of Pharmacy, in speaking upon the adulteration of drugs purchased by him in the open market, says: "Of five samples of olive oil, four of them were found to be cottonseed oil, the fifth was composed of about equal parts of green olive oil and cottonseed oil. Two samples of honey purchased of pharmacists were found to be pure, while a third, from a grocer, was nearly all glucose. Of seven samples of spirits of nitrous ether examined by the gasometric method of the *U. S. Pharmacopœia*, not one was found that could be said to approach the official strength. Three samples were absolutely lacking in ethyl nitrite, and the remaining four gave results as follows: 2.60, 2.45, 2.03, and 1.90 per cent." "There is no valid excuse for this," says Prof. Davoll, "as we have been told many times how this important pharmaceutical product may be prepared so as to keep a reasonable length of time. It should be made often, in small amount, and properly preserved. Occasional testing may be made with practically no expense. The *U. S. Pharmacopœia* method of assay is of easy execution. Some cochineal bugs examined yielded 68 per cent. of ash, when the *Pharmacopœia* allows but 5 per cent. Most of this was found to be barium sulphite.

Quinichloral.—This is a mixture of quinine and chloral, and is said to be superior to either drug alone. It is recommended as a substitute for the metallic antiseptics, and is said to destroy bacteria more readily than corrosive sublimate. It is a thick, oily liquid, soluble in water and alcohol.

Purified Coal Tar.—Commercial coal tar may be purified for medicinal use by dissolving in three times its weight in benzine, filtering and evaporating, or distilling of the solvent. The residue mixes readily with vaseline, lanoline, etc.—*Pharm. Cent.*

To Tell the Age of Milk.—Dr. Vaudin has found (*Bull. de l'Acad. de Med.*) that if a few drops of a solution of indigo-carmine are added to milk the color produced by it disappears under the action of the microbes in the milk. He determines the age of the milk by the duration of the tint; thus, in fresh milk it lasts about twelve hours at 15° C., five hours at 15° to 20° C., and four hours at 20° C. When there are several decigrammes of lactic acid to the quart of milk, the tint vanishes almost instantaneously. This, of course, can apply only to milk not protected by an antiferment.

Detection of Formaldehyde in Milk.—It is said that if from 1 to 2 c.c. of a 1 per cent. solution of phloroglucin be added, together with one drop of solution of potassa to 10 c.c. of milk, a red color is at once developed if traces of formic aldehyde are present. This is said to form an exceedingly delicate reagent for the detection of this preservative.

Doses in the U. S. Pharmacopœia.—The medical profession is making strenuous efforts to have the next revision of this work contain the maximum and minimum doses of drugs.

Testing the Purity of Chloroform.—Dr. Leucke, of Strasburg, gives the following simple method of testing the purity of chloroform : Immerse a small piece of thin white blotting paper in the chloroform, and then let it dry in the air. As soon as the chloroform has evaporated the paper will not present the least smell if the chloroform is pure. If there is any rancid smell perceptible it indicates the presence of butyric acid in the chloroform, and, as a rule, is the strong characteristic of that substance.—*Philadelphia Medical Times.*

Milk as a Culture Medium.—According to J. W. Strickler (*Philadelphia Medical Journal*), the composition of milk makes it a nutritive medium for every germ whose biology is at present understood. An investigation carried on in Boston showed that when healthy cows, in a clean place, were carefully milked into a sterilized flask the milk contained only 530 bacteria per cubic centimetre ; but when the ordinary milk-pail is used and the milk is conducted in the common way there were on an average 30,500 bacteria per cubic centimetre immediately after milking. It should be remembered that the germs of diphtheria, cholera, typhoid fever, and scarlet fever develop at ordinary room-temperature, and that such growth does not affect the gross appearance of the milk.

Review of Tuberculin Trials.—Dr. G. D. Head, in the *St. Paul Medical Journal* for September, collects the results of the work of those who have used tuberculin for diagnostic purposes in human practice, and also gives in detail the clinical experiments made by himself. "As it is now recognized," says the *Medical Standard*, "that a large percentage of the cases of tuberculosis in man can be cured if diagnosed sufficiently early, the importance of a method which promises to do this is certainly a matter of vital interest."

Dr. Head says there is a lack of uniformity among different observers as to the amount of tuberculin which should be used and the number of injections which should be employed. Some use three or four injections at intervals of three to seven days, commencing with small doses, which are gradually increased. Others give much larger doses from the first. A third class, to which Dr. Head belongs, give the tuberculin in a single dose of moderate size—10 mgr.

"Tuberculin produces little or no disturbance when injected into a healthy individual unless it is given in very large doses. In persons infected with tubercle bacilli it produces a train of well-marked symptoms.

"An individual is said to react to tuberculin when he develops within twelve hours following the injection a rise in temperature of 1.5° to 2° or more above the mean course of the temperature previous to the injection. Other symptoms, such as headache, malaise, anorexia, and sometimes vomiting and diarrhœa, accompany the temperature rise, but they are not the distinctive features of the reaction.

"Some patients complain of pain in the chest, usually upon one side. In some cases the cough is increased and more sputum is expectorated, which may contain blood. In the lungs Bertheusen has observed increased areas of dulness. Sibilant and moist râles are usually heard in increased number in the involved area of the lungs, and sometimes in areas where before no abnormal sounds could be detected."

Dr. Head has been able to collect 487 recorded cases of tuberculin injection, given for diagnostic purposes. "This represents the work of nine observers. Of this number 266, or 54 per cent., reacted, and 221, or 46 per cent., did not react. When one considers that in general suspected cases were injected, the tuberculin does not show too large a percentage of individuals infected with the disease. In all those cases where records were made, namely, 136 reactions, 83, or 61 per cent., were demonstrated as tuberculous by post-mortem, operative, or bacteriological means.

Of the remaining, some cases were lost track of, some died without post-mortem examination, and some were clinically diagnosticated as tuberculous, but the diagnosis was not verified."

In Dr. Head's series of cases, 69 per cent. of those reacting were demonstrated as tuberculous by the finding of the tubercle bacilli in the sputum or feces. Add to these figures the evidence offered by the veterinarians, that 99 per cent. of cattle reacting are demonstrated as tuberculous at autopsy, and no more convincing argument can be offered in favor of tuberculin as a diagnostic agent.

A further study of the statistics gives an answer to other interesting questions:

"1. Do all surely tuberculous individuals react to the tuberculin?"

"Of the 83 cases undoubtedly tuberculous, 4, or nearly 5 per cent., did not react to the tuberculin injection. So far as the writer has been able to consult the original reports, all these cases were far advanced in the disease, when a diagnosis by other means was easy and certain, and the tuberculin would not be required to establish the nature of the disease. We, therefore, consider it demonstrated that a small number, say 5 per cent., of all cases of tuberculosis far advanced in the disease do not react to the tuberculin. Whether or not such cases would have reacted in the incipient stage of the infection cannot now be answered.

"2. What percentage of apparently healthy individuals in whom no evidence of tuberculosis can be detected react to tuberculin?"

The author's personal investigation resulted as follows: Of the 47 persons injected, 12 were apparently healthy, normal individuals, so far as physical examination and general appearance could determine; of this number one reacted, or about 8 per cent. All were given 0.24 c.c. of Bureau tuberculin. Maragliano is reported as having obtained a reaction in 9 per cent. and Guttsdat in 8 per cent. of apparently healthy individuals.

The author's inference is that the reaction may be due to bacilli which may be encapsulated in the apices of the lungs or bronchial glands. Sooner or later in most cases the barrier of the cells will break down and other areas will be involved.

"3. How does tuberculin act upon individuals affected with diseases supposedly non-tuberculous in character?"

In 64 cases which were injected, 18 per cent. gave a reaction. Injections were made in a wide range of diseases, both acute and chronic. "Cases in which reactions were obtained were reported as follows: One case of lateral sclerosis, spastic paraplegia, cerebrospinal meningitis, chlorosis, syphilis, leprosy, sarcoma of the pharynx, hydrarthrosis, suppurating inguinal glands, acute articular rheumatism, and two cases of articular rheumatism.

From the evidence it seemed probable that in no one disease, aside from tuberculosis, does tuberculin cause a reaction in any marked percentage of cases, if at all. It is most likely that the cases clinically diagnosed non-tubercular and reacting were in patients harboring a slight latent tubercular infection.

"4. What is the effect of tuberculin upon those diseases in which the tubercle bacillus is acknowledged to be the sole or prominent etiological factor?"

In 39 cases of pulmonary tuberculosis, in all of which bacilli were found in the sputum, 36, or 92 per cent., reacted. In 35 cases of enlarged cervical glands of the neck, 24, or 71 per cent., reacted. In 18 cases of acute pleurisy, 16, or 88 per cent, reacted. In 3 cases of chronic pleurisy 100 per cent. reacted. In 24 cases of tuberculosis of the joints, 22, or 91 per cent., reacted. In 9 cases of tubercular peritonitis, 100 per cent. reacted. In 2 cases of lupus all reacted. In 2 cases of Addison's disease all reacted.

Such results as these are incontrovertible evidence in favor of the diagnostic value of tuberculin reaction.

Origin of Calomel.—Calomel was discovered by Crollious in the seventeenth century, and the first directions for its preparation were given by Beguin in 1608. Its name is derived from two Greek words signifying "a beautiful black," because in its preparation a black powder is the first step in the manufacture, being produced by rubbing mercury together with corrosive sublimate.

An Experiment in Immunity.—Dr. Ewart, Professor of Natural History at Edinburgh, has been devising breeding experiments which are of the greatest theoretical and practical interest. In one of these experiments it was thought that zebra mules might possibly prove to be immune to the disease communicated by the Tsetse fly, and if so, the problem of African transport might be solved. Professor Ewart inoculated three of his hybrids with the Tsetse fly, but, unfortunately, it turns out that the inoculated animals, though more resistant than horses, all died in about eight weeks.

